# Worldwide Evaluations of Quinoa: Preliminary Results from Post International Year of Quinoa FAO Projects in Nine Countries

**DOI:** 10.3389/fpls.2016.00850

**Published:** 2016-06-21

**Authors:** Didier Bazile, Cataldo Pulvento, Alexis Verniau, Mohammad S. Al-Nusairi, Djibi Ba, Joelle Breidy, Layth Hassan, Maarouf I. Mohammed, Omurbek Mambetov, Munira Otambekova, Niaz Ali Sepahvand, Amr Shams, Djamel Souici, Khaled Miri, Stefano Padulosi

**Affiliations:** ^1^Unité Propre de Recherche Gestion des Ressources Renouvelables et Environnement, Department of Environments and Societies, French Agricultural Research and International Cooperation OrganizationMontpellier, France; ^2^CNR–Institute for Agricultural and Forest Systems in the MediterraneanErcolano, Italy; ^3^ESA–Ecole Supérieur d'AgricultureAngers, France; ^4^General Seed Multiplication Cooperation, Agricultural Research and Extension AuthorityDhamar, Yemen; ^5^Centre National de Recherche Agronomique et de Développement AgricoleKaedi, Mauritania; ^6^Department of Plant Breeding, Lebanese Agricultural Research InstituteZahlé, Lebanon; ^7^Plant Production Department, Ministry of AgricultureBaghdad, Iraq; ^8^Genetics and Plant Breeding, Forage and Range Research Program, Agricultural Research CorporationKhartoum North, Sudan; ^9^Seed Association of KyrgyzstanBishkek, Kyrgyz; ^10^Seed Association of TajikistanDushanbe, Tajikistan; ^11^Research Department, Seed and Plant Improvement InstituteKaraj, Iran; ^12^Crop Intensification Research Department, Field Crops Research Institute, Agricultural Research CenterGiza, Egypt; ^13^Département Recherche Expérimentation, Institut Technique de Développement de l'Agronomie SaharienneBiskra, Algeria; ^14^Bioversity InternationalMaccarese, Italy

**Keywords:** *Chenopodium quinoa* Willd., plant genetic resources, seeds, adaptation, climate change, multi-local trials, agrobiodiversity, agroecology

## Abstract

*Chenopodium quinoa* Willd., a high quality grain crop, is resistant to abiotic stresses (drought, cold, and salt) and offers an optimal source of protein. Quinoa represents a symbol of crop genetic diversity across the Andean region. In recent years, this crop has undergone a major expansion outside its countries of origin. The activities carried out within the framework of the International Year of Quinoa provided a great contribution to raise awareness on the multiple benefits of quinoa as well as to its wider cultivation at the global level. FAO is actively involved in promoting and evaluating the cultivation of quinoa in 26 countries outside the Andean region with the aim to strengthen food and nutrition security. The main goal of this research is to evaluate the adaptability of selected quinoa genotypes under different environments outside the Andean region. This paper presents the preliminary results from nine countries. Field evaluations were conducted during 2013/2014 and 2014/2015 in Asia (Kyrgyzstan and Tajikistan), and the Near East and North African countries (Algeria, Egypt, Iraq, Iran, Lebanon, Mauritania, and Yemen). In each country, the trials were carried out in different locations that globally represent the diversity of 19 agrarian systems under different agro-ecological conditions. Twenty-one genotypes of quinoa were tested using the same experimental protocol in all locations consisting in a randomized complete block design (RCBD) with three replicates. Some genotypes showed higher yields and the Q18 and Q12 landraces displayed greater adaptation than others to new environmental conditions. The Q21 and Q26 landraces were evaluated with stable and satisfactory levels of yield (>1 t.ha^−1^) in each of the different trial sites. This production stability is of considerable importance especially under climate change uncertainty. While these results suggest that this Andean crop is able to grow in many different environments, social, and cultural considerations remain crucial regarding its possible introduction as a staple food in new cropping systems around the world.

## Introduction

*Chenopodium quinoa* Willd. is a plant is a plant originated in the Andean Plateau, around Lake Titicaca, 3800 m above sea level (m.a.s.l.) on the Peruvian-Bolivian border (Heiser and Nelson, 1974; Jacobsen, 2003). Quinoa domestication began there about 7000 years ago (Bazile et al., 2013), and brought a significant increase in the genetic diversity of the species cultivated (Bhargava et al., 2007a,b). Such a high genetic diversity in quinoa is closely related to the vastness of its center of origin and the varied of human uses that have been influencing the selection process over time (Bazile and Negrete, 2009). This is the case with many domestication processes of numerous important crops and their wild relatives (Wilson, 1990). The selection exerted by local communities has led to many landraces, which can be still found grown especially in Bolivia and Peru (Risi and Galwey, 1984; Bazile et al., 2014; Bazile, 2015).

At global level, there are more than 6000 varieties of quinoa cultivated by farmers (Rojas et al., 2015). Those varieties can be classified into five main categories or ecotypes, according to their adaptation to specific agro-ecological conditions in major production areas (Bois et al., 2006; Rojas, 2003; Anabalón and Thomet-Isla, 2009; Fuentes et al., 2009a; Fuentes and Bhargava, 2011; Bazile et al., 2013, 2014). Quinoa of the inter-Andean valleys grows in areas between 2300 and 3500 m.a.s.l., characterized by annual rainfall between 700 and 1500 mm. Quinoa grows in highlands (also called *Altiplano* of the Andes) between 3500 and 3900 m.a.s.l. in areas with an annual rainfall of 400–800 mm. Quinoa from the edges of deserts and high altitude salt lakes (*Salares*) grows in areas nearly 4000 m.a.s.l., characterized by a limited volume of annual rainfall (150–300 mm) and with many days of frost. Quinoa found at sea level (Coastal) is adapted to the regions lying between sea level and 1000 m.a.s.l., where annual rainfall ranges from 500 to 1500 mm (Martínez et al., 2009). Quinoa from the *Yungas* grows under tropical moisture conditions and in areas with high levels of precipitations.

Considering the high genetic diversity of quinoa, the needs of the crop vary extensively by landrace or cultivar (Cleveland et al., 1994; Brookfield et al., 2002; Chevassus-au-Louis and Bazile, 2008). Due to the diverse characteristics of the five ecotypes, quinoa can be grown under very different climatic conditions (Jacobsen, 1997; Jacobsen et al., 2003; Christensen et al., 2007; Fuentes et al., 2009b, 2012). Sandy loam soils with good drainage and if possible, a high content of organic matter and nutrients are preferable for quinoa to better adapt to new environments. It is also advisable to work in neutral soils, although quinoa can tolerate different pH and grow in alkaline (to pH 9) and acid soils as well (up to pH 4.5; Narea, 1976; Tapia, 1979). As previously described, quinoa cultivation can be pursued in many climatic conditions, including desert, hot, dry, cold and dry, temperate, and rainy or hot with high humidity (Bosque et al., 2003; Gesinski, 2008). Indeed, scientific evidence exist to confirm that quinoa tolerates very dry conditions and drought: quinoa uses water very effectively, even though it is a C3 plant, due to physiological mechanisms (Cocozza et al., 2013) that allow the plant to prevent moisture deficits, and tolerate and/or withstand lack of soil moisture. An ideal average temperature for quinoa would be around 15–20°C, but some specific landraces can also withstand extreme temperatures from −8°C to +38°C (Bazile et al., 2015). Sensitivity periods to temperatures have been recorded mainly when seeds germination occurs in cold temperatures (frost) and when flowering takes place under high temperatures (FAO, 2011). Quinoa is able to endure extreme solar radiation, allowing it to store hours of heat necessary to carry out its vegetative and productive phases. Quinoa is cultivated in areas from 2° North latitude to 47° South latitude, from Colombia to Chile in South America.

There are varieties adapted to short days, long days and those photoperiod insensitive (Bertero et al., 1999; Bertero, 2001). There are two major phases in the development of quinoa plants: the vegetative phase is an active growth phase during which the plant acquires new properties to reach vegetative maturity. This is followed by the reproductive phase, which is the period when the plant will be able to produce flowers and seeds and reach physiological maturity. Depending on the photoperiod sensibility of each variety, each stage's duration can be modified depending on the length of days and on temperatures (Risi and Galwey, 1989, 1991; Jacobsen and Stølen, 1993). Photoperiod sensitivity is a key factor in the adaptation of this crop at new latitudes (Bertero, 2001).

For thousands of years, quinoa has been a staple food for Andean populations (Tagle and Planella, 2002; Planella et al., 2011; Martinez et al., 2015). Mainly used as a cereal grain (it is in many cases considered a pseudo-cereal), botanically speaking quinoa is an achene, a seed-like fruit with a hard coat (Cusack, 1984; NRC, 1989). Classified as a member of the *Amaranthaceae* (a large family of 160 genera and 2400 species- Sing, 2010) from the genus *Chenopodium*, the specie *C. quinoa* Willdenow is gaining importance particularly for its high and well-balanced nutritional contents. Quinoa has exceptional nutritional properties, with high protein content in comparison to cereals, which is combined with a good balance of essential amino acids (Vega-Gálvez et al., 2010; Maureira and Martínez, 2012; Miranda et al., 2012; Lutz et al., 2013). The quinoa crop has recently undergone important developments around the world regarding its ability to withstand extreme conditions (Bazile et al., 2015). Its high genetic diversity provides opportunities for leveraging its hardiness and further its wide adaptation (Louafi et al., 2013). Many countries regularly face food insecurity problems, often combined with a difficult agricultural environment (FAO, 2015). Today, drought and soil salinization are major limiting factors in cultivation, a fact that is generating significant pressure on arable land availability. Considering these major challenges, quinoa hardiness is increasingly being appreciated by growers: today the crop is presently cultivated, or is under experimentation, in more than 95 countries and its cultivation continue to expand rapidly worldwide (Bazile, 2015; Bazile et al., 2016).

The main quinoa producers in the world are Bolivia, Peru, Ecuador, and the United States of America. In 2013, over 75,000 hectares of land were under quinoa cultivation in Bolivia and more than 45,000 hectares in Peru. These two countries are still the major producers in the Andes and in the world. Today the cultivation of quinoa has reached countries as far as Tibet, Morocco, France, India, China, the United Kingdom, Sweden, Denmark, Netherlands, and Italy, among others (Bhargava et al., 2006; Pulvento et al., 2010; Bazile, 2015; Bazile et al., 2015). From the 1950s to nowadays, trials for plant breeding and/or crop adaptation have been conducted in Andean countries but also in other parts of the world in order to better understand the domestication process of quinoa and to obtain quinoa germplasm adapted to new environmental conditions (Bonifacio et al., 2015; Jellen et al., 2015). Quinoa is a viable alternative for food insecure countries in a world facing increasingly climate challenges and set to feed a growing population in terms of both food and nutrition security (Galwey, 1992; Ruiz et al., 2014).

The FAO project “American and European Test of Quinoa” (1996–1998) was the first mechanism for the diffusion of quinoa worldwide and underlies the current global expansion of the crop (Mujica et al., 2001). Field trials were established in several countries to evaluate the performance of quinoa through multiple experiments at the international level. Since 1996, quinoa has been recognized as one of the most promising crops in terms of food security (Schlick and Bubenheim, 1996). While the major producers are still located in the Andean region, quinoa cultivation is a reality across all continents where germplasm originated from the Andes was successfully selected (Bazile et al., 2016).

The first objective of the International Year of Quinoa (IYQ) in 2013 was to increase the visibility of the great potential of quinoa biodiversity to contribute to global food security, especially in countries where the population has no access to other protein sources or where production conditions are limiting. In many of the countries in North Africa and the Near East, food security still remains a major problem for vulnerable population groups. Global agricultural production is facing problems that threaten its stability and sustainability, such as climate change, land salinization, limited water availability for agriculture (Gómez-Pando et al., 2010). Its high tolerance to extreme weather conditions and high nutritional value contributed to choosing quinoa as a potential crop to address these challenges (Delatorre-Herrera and Pinto, 2009; Orsini et al., 2011; Ruiz-Carrasco et al., 2011; Pulvento et al., 2012). Following the IYQ, the Food and Agriculture Organization of the United Nations (FAO) initiated a multi local test (through FAO-TCP for Technical Cooperation Programmes) in a number of countries of North Africa, the Near East and Asia viz. Algeria, Egypt, Iran, Iraq, Kyrgyzstan, Lebanon, Mauritania, Sudan, Tajikistan, and Yemen. During the implementation of these projects, FAO collaborated with many partners worldwide to access quinoa seeds and their expertise in the field of crop cultivation. Various research centers, universities and seed firms were mobilized to find quinoa seeds of different varieties to be tested. Twenty different genotypes were eventually made available to countries for the international trial programme.

The main goal of this research was to evaluate the adaptability of selected quinoa genotypes under different environments outside the Andean region. Two hypotheses have been put forward in this paper to assess the performance of quinoa genotypes in new environments viz: Hypothesis (1): “*Within the high genetic diversity of the species, it is possible to identify the most suitable variety for each study site*” and Hypothesis (2): “*To meet the various uncertainties related to global change in each region, yield stability of one variety across all study sites represents an important indication of genotype suitability and decreased risks when cultivating it any other given area*.” Analysis of these two hypotheses will structure the discussion on two possible pathways: yield maximization for potential production and yield optimization for production stability (Tilman et al., 2002).

## Materials and methods

### Study sites

The paper concerns an analysis of the results from nine countries with similar ecological conditions. In this set, countries are located in North Africa, the Near East, and Asia under quite similar semi-arid or arid climatic conditions, namely in Algeria, Egypt, Iran, Iraq, Kyrgyzstan, Lebanon, Mauritania, Tajikistan, and Yemen.

**Figure 1:**
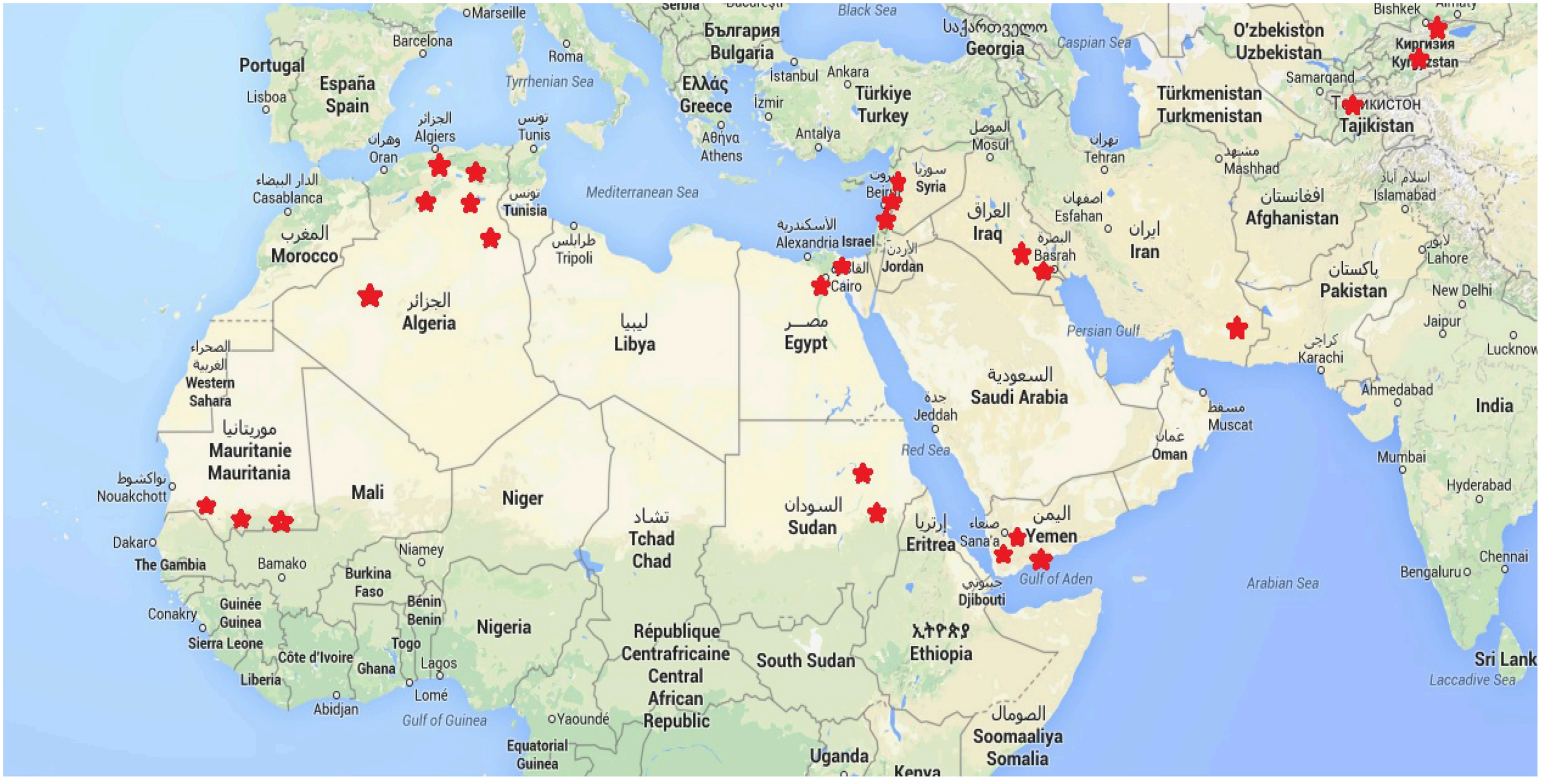
**Location of the study sites**.

Within each of these countries, 2–5 sites were chosen to represent the diversity of agro-ecological zones at country level. The study sites are parts of the decentralized network of agronomic stations from each of the National Research Centers involved in the programme. We tested a set of 21 different quinoa genotypes and their responses in terms of agronomic performance cultivation in these contrasted environments were duly recorded. The locations of these sites are found in Figure 1. Table 1 also provides the principal descriptive data of each of 19 sites surveyed and used for this paper. The sowing dates reflect ecological and agronomic differences across study sites to adapt quinoa cycle into local cropping systems (Table 1).

**Table 1:** **General characteristics of the study sites**.

**Country**	**Study Site**	**Site name**	**ALT**	**LAT**	**LONG**	**Climate**	**Soil**	**pH**	**Average annual rainfall**	**Average annual temperature**	**ETP**	**Quinoa sowing date**
Algeria	AL1	Biskra	270	34.3	5.38	Arid	Clay-Loam	8.2	124	21.7	2144	22/09/2014
Algeria	AL2	El-oued	25	33.7	6	Saharian	Sandy-Loam	7.9	50	28	2107	22/09/2014
Iraq	IQ1	Al-qurna	2	30.94	47.45	Arid	Clay-Loam	7.8	130	25.07	2121	19/12/2013
Iraq	IQ2	Al diwaniyah	24	32.01	44.89	Arid	Clay-Loam	7.8	120	24.48	1911	17/12/2013
Yemen	YE1	Ta'izz	1300	13.3	44.1	Temperate	Clay-Sandy-Loam	8	175	26	1700	07/01/2014
Yemen	YE2	Al-kadan	252	12.42	45	Tropical	Clay-Sandy	8.3	175	30	2100	09/01/2014
Yemen	YE3	Dhamar	2700	14.55	44.4	Temperate	Clay-Loam	7.8	225	18	1300	07/01/2014
Egypt	EG1	Ismailia	13	30.06	32.2	Mediteranean	Sandy	7.8	16	21.9	1800	15/12/2013
Egypt	EG2	Giza	19	30.01	31.22	Mediteranean	Clay	7.8	9.2	20.8	1800	16/12/2013
Iran	IN3	Iranshahr	591	27.2	60.7	Saharian	Reg Soil-Hamada	7.8	113	26.5	1500	24/12/2013
Lebanon	LB1	Tyr	5	33.15	35.13	Mediterranean	Sandy Calcareous	8.9	700	20	1200	26/12/2013
Lebanon	LB2	Tel-amara	925	33.51	35.59	Semi-arid Continental	Clay	7.9	550	15	1500	04/08/2014
Lebanon	LB3	Tarchich	1036	34.88	36.72	Arid continental	Clay-Loam	7.6	350	14	1650	16/05/2014
Mauritania	MA1	Kankossa	51	15.92	−15.52	Saharian	Sandy	7.8	600	29	2400	30/12/2013
Mauritania	MA2	Rindao	11	16.14	−13.58	Saharian	Loam	7.6	250	29	2400	23/12/2013
Mauritania	MA3	Dieri	21	15.52	−12.99	Saharian	Sandy	7.6	250	29	2400	13/01/2014
Tadjikistan	TA1	Mukarramov	1200	36	67	Semi-arid Continental	Clay-Loam	7.8	250	25	1200	06/05/2014
Kyrgyzstan	KY1	Suzak	700	40.5	73.55	Continental	Stained Clay Light Gray	7.4	285	12.3	1406	01/05/2014
Kyrgyzstan	KY2	Chui	1150	42.45	74.3	Continental	Stained Clay Gray	7.8	375	10.1	950	30/04/2014

### Genetic resources materials

In Table 2, the different genotypes used were classified in three groups considering a gradient of the level of genetic diversity among them, from landraces (heterogeneous crop population varieties) to varieties under development (still conserving a degree of heterogeneity) and improved varieties (homogeneous plants) protected by intellectual property rights (Plant Variety Protection, PVP; Bazile et al., 2016).

**Table 2:** **Classification of the 21 quinoa genotypes used for trials**.

**10 Landraces**	**8 Varieties under development**	**3 Improved varieties (Registered with plant variety protection)**
Q12 (Chile)	Sajama (Bolivia)	Regalona (Chile)
Q18 (Chile)	Santamaria (Bolivia)	Puno (Denmark)
Q19 (Chile)	Q1 (U.A.E)	Titicaca (Denmark)
Q21 (Chile)	Q2 (U.A.E)	
Q22 (Chile)	Q3 (U.A.E)	
Q26 (Chile)	GIZA 1 (Egypt)	
Q27 (Chile)	GIZA 2 (Egypt)	
Q29 (Chile)	SAJAMA Iranshar (Iran)	
Q31 (Chile)		
Quinoa real (Bolivia)		

Due to the difficulties to access quinoa germplasm at global level, each country made specific requests through its networks. FAO has mobilized various partnerships to collect different quinoa accessions to carry out the study. First, through a collaboration with the International Center for Biosaline Agriculture (ICBA, a non-profit International Organization)—a research center based in the United Arab Emirates (UAE)—FAO could access specific varieties of quinoa. Seeds of the three varieties under development (Q1, Q2, and Q3) were obtained from ICBA to be made available for the trials in Yemen. FAO has also received seeds from the *Centro di Ricerca per la Cerealicoltura* (CRA-CER) in Italy which helped to expand the genotypes proposed in the tests. The seeds of nine accessions of landraces (Q12 to Q31) were obtained from CRA-CER who has been working and selecting these accessions in Italy after accessing them from the United States Department of Agriculture genebanks (USDA). These seeds had Chilean origin. Seeds were supplied to FAO-RNE which has distributed them to eight countries in the region. The two quinoa varieties (Sajama and Santamaria) were provided by PROINPA in Bolivia to the Seed and Plant Improvement Institute (SPII) in Iran. Selected seeds of early matured plants from the genotype “Sajama” have produced a new variety that was called “Iranshahr.” Giza1 and Giza2 have been selected in Egypt from preliminary quinoa lines furnished by the University of Copenhagen in Denmark. Finally, *Puno* and *Titicaca* are two varieties of the Quinoa Quality Enterprise linked to the University of Copenhagen (Denmark). *Regalona* is the only quinoa variety with PVP developed by Von Baer Seeds for the Southern part of Chile (Von Baer et al., 2009). Finally, each country could choose from over 21 fairly differentiated genotypes.

### Experimental protocol

Given the specificities of the FAO TCP projects, each country independently developed its experiments with similar technical support provided at regional level. Due to the difficulties of accessing quinoa genetic resources for some countries and in order to avoid biases in the analysis, a senior statistician provided advice on how to overcome such gaps (not all the countries have tested the same genotypes) and how to define and conduct solid statistical analyses in order to obtain scientifically reliable results. To be able to do that, in each test, the experimental protocol was based on a common core curriculum. For each site, the parameters used were described in the Tables 1, **4**.

The experimental design, a randomized complete block design (RCBD) with three replicates, represents the core protocol. The distance between two blocks was more than 1 m. Each of replicate plots is of the same area, i.e., 5 m long, 2 m wide, 10 m^2^. The replicate plot had four rows of plants to define an inter-rank of 50 cm and a 25 cm distance between plants. In addition, 3–5 seeds were sown per hole of about 1 cm depth that was covered later. In few cases, irrigation was to secure germination step. Fifteen days after sowing, considering the germination rate, two plants were kept per hole by eliminating the other and considered for the experiment.

It is however important to note that the different varietal types (landraces, varieties under development, and improved varieties) were not necessarily used in each trial. On some sites, only landraces were tested, and other tests were only conducted with improved varieties (see Tables 2, 3). These differences are explained by the difficulties to access germplasm and by the specific demands of each country. The resulting incompleteness of the experimental design has oriented our statistical analysis below.

**Table 3:** **Distribution of the genotypes used for trials in the experiment plan**.

**Study sites by country**			**Genotypes**																					
			**SANTA MARIA**	**SAJAMA**	**Q12**	**Q18**	**Q19**	**Q21**	**Q22**	**Q26**	**Q27**	**Q29**	**Q31**	**QUINOA REAL**	**Q1**	**Q2**	**Q3**	**IRANSHAHR**	**REGALONA**	**PUNO**	**TITICACA**	**GIZA1**	**GIZA2**	**TOTAL Genotypes**
ALGERIA																								
	AL1		1	1	1	1		1	1	1	1	1										1	1	***11***
	AL2		1	1	1	1		1	1	1	1	1										1	1	***11***
EGYPT																								
	EG1		1	1	1	1	1	1	1	1	1	1	1									1	1	***13***
	EG2		1	1	1	1	1	1	1	1	1	1	1									1	1	***13***
IRAN																								
	IN3		1	1	1	1	1	1	1	1	1	1	1					1			1			***13***
IRAQ																								
	IQ1				1	1	1	1	1	1	1	1	1											***9***
	IQ2				1	1	1	1	1	1	1	1	1											***9***
KYRGYZSTAN																								
	KY1																		1	1	1			***3***
	KY2																		1	1	1			***3***
LEBANON																								
	LB1		1	1	1	1	1	1	1	1	1	1	1											***11***
	LB2		1	1	1	1	1	1	1	1	1	1	1									1	1	***13***
	LB3		1	1	1	1	1	1	1	1	1	1	1									1	1	***13***
MAURITANIA																								
	MA1		1	1	1	1	1	1	1	1	1	1	1	1										***12***
	MA2		1	1	1	1	1	1	1	1	1	1	1	1										***12***
	MA3		1	1	1	1	1	1	1	1	1	1	1	1										***12***
TADJIKISTAN																								
	TA1																		1	1	1			***3***
YEMEN																								
	YE1		1	1	1	1	1	1	1	1	1	1	1		1	1	1							***14***
	YE2		1	1	1	1	1	1	1	1	1	1	1		1	1	1							***14***
	YE3		1	1	1	1	1	1	1	1	1	1	1		1	1	1							***14***
TOTAL Sites		14	14	16	16	14	16	16	16	16	16	14	3	3	3	3	1	3	3	4	6	6	

(“1” = presence).

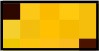
 Subset (16 sites × 11 genotypes) extracted from the whole data set to a posteriori generate an experiment plan that is almost complete for the ANOVA.

Table 4 shows an example of a spreadsheet used to collect and measure data in the experimentation using the international descriptor list for quinoa (Bioversity International et al., 2013).

**Table 4:** **Example of a spreadsheet to collect data in the field**.

**Country:**				
**Study site:**				
**Varieties**	**Genotype 1**			
**Data observations/measures**	**R1**	**R2**	**R3**	**Average**
Date of Sowing				
Days to 2–8 true leaves				
Days to budding stage				
Days to the beginning of flowering				
Days to 50% flowering				
Days to maturity				
Date of harvesting				
No. days from planting to harvest				
Plant height (cm)				
Number of branches/plant				
1000-seed weight (g)				
Weight of the main head (g)				
Width of panicle (cm)				
Length of panicle (cm)				
Panicle color				
Germination Rate (%)				
Number of plants on the harvest area (nb/m2)				
Seed yield/plant (g)				
Seed yield/ha (ton)				

Pre-treating the seed with an insecticide and a fungicide was done before the test only, whenever such problems were found to exist in the area. In terms of soil preparation, plowing was performed by a weedier harrow to make the seedbed as thin as possible and facilitate soil-seed contact given the small diameter of the seeds.

### Statistical analysis

Estimates Yields were based on the whole plots (10 m^2^). As part of this study, two different averages were used to compare performances between genotypes and sites: the arithmetic average and the weighted average. Weighted average is important when you are dealing with frequencies or distributions. In our case, each site does not have the same number of genotypes studied, so the averages were weighted by the number of accessions used in the selected site to obtain actual weighted averages, reflecting more appropriately the results of the study for comparisons.

Considering the reality of our global experiment plan, the analysis of variance was only applied to an incomplete ANOVA table referring to some selected sites where similar genotypes were used extensively. A subset (16 sites × 11 genotypes) was extracted from the whole data set to a posteriori generate an experiment plan that is almost complete (Table 3).

A two-way analysis of variance was performed with the R Software to test for differences in yield across sites and genotypes. No site-genotype interaction was included in the model because of a lack of repetitions in some sites. Yield was square-rooted transformed prior to the analysis to stabilize the variance (See Supplementary Material). A one-way analysis of variance was also conducted to test for differences in square-rooted yield across sites, leaving the between-genotype variability in the residual variance. The analysis was complemented with a multiple comparison test using Tuckey's method. The same analysis was conducted using genotype instead of site as the predictor.

## Results

Yield was significantly different across sites and genotypes (two-way ANOVA: F-statistic for the site effect: 43.0, *p* < 0.001, F-statistic for the genotype effect: 11.9, *p* < 0.001).

**Figure 2:**
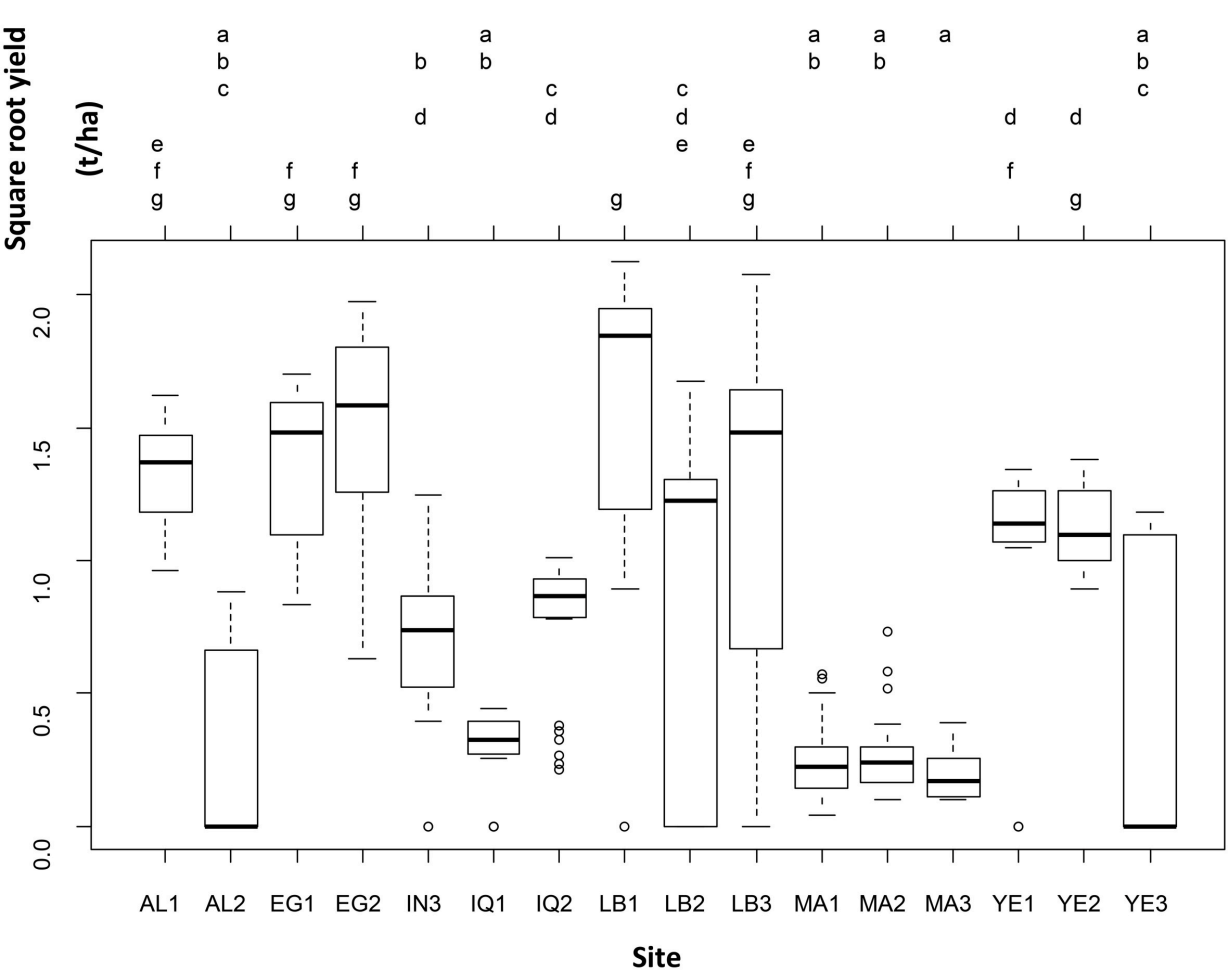
**Results of the one-way analysis of variance conducted to test for differences in square-root yield across sites, leaving the between-genotype variability in the residual variance**.

Even when leaving the genotype variability into the residual error, yield was significantly different across sites (one-way ANOVA: F-statistic: 30.1, *p* < 0.001). Seven groups of sites out of sixteen were distinguished on the basis of yield (Figure 2).

**Figure 3:**
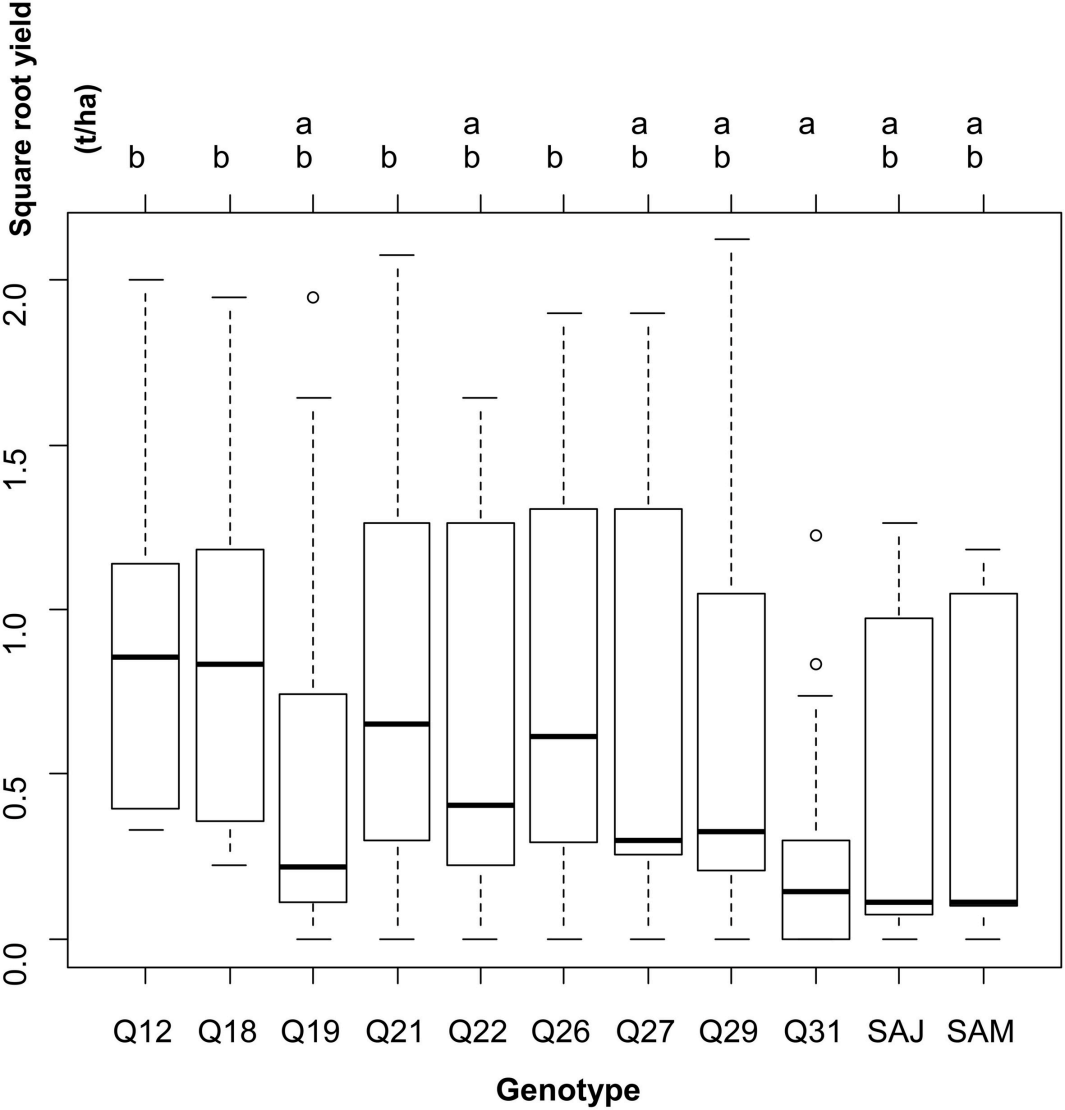
**Results of the one-way analysis of variance conducted to test for differences in square-root yield across genotypes, leaving the between-site variability in the residual variance**.

Reciprocally, when leaving the site variability into the residual error, yield was significantly different across genotypes (one-way ANOVA: F-statistic: 3.3, *p* < 0.001). Two groups of genotypes were distinguished on the basis of yield (Figure 3).

### Yield performances per site for all the varieties tested

Table 3 and Figure 2 show that there are significant differences in yield among genotypes tested in different countries. A weighted average yield of 1.07 t.ha^−1^ was obtained for all varieties and all countries combined. The results demonstrate that quinoa can be adapted in many environments while having higher yields than those that can be obtained in Bolivia or Peru (about 1 t.ha^−1^). Considering the strong differences between varieties and countries, it is then necessary to investigate more in depth the results of each of the countries involved in the project. The Tuckey test highlights a large gradient of yields across the study sites from LB1 (group g) to MA3 (group a; Figure 2). The results by country are described below but we can underline here that the higher yields were obtained at LB1 (g), EG1 and EG2 (f, g), and AL1 and LB3 (e, f, g). In addition, MA3 (a), MA1 and MA2 and IQ1 (a,b) and, AL2 and YE3 (a, b, c) did not performed well and yields were always lower than in other locations.

#### Algeria

Two sites were analyzed and 13 genotypes were tested (Table 3). The yield average obtained was 1.65 t.ha^−1^ at the first site (AL1) and 0.26 t.ha^−1^ at the second site (AL2) which gives an arithmetic average of 0.96 t.ha^−1^ for the country. Note that the lower values at the second site, never exceeding 0.78 t.ha^−1^, contributed to the fact that the arithmetic average of the country remains below the value of the weighted average yield for all countries. The three genotypes Q26 (2.62 t.ha^−1^), Q18 (2.27 t.ha^−1^), and Q27 (2.17 t.ha^−1^) achieved the much higher yields at the first site.

#### Egypt

Two sites were analyzed and 13 genotypes were tested (Table 3). A higher value than the weighted average yield for all countries was obtained with 1.89 t.ha^−1^ at the first site (EG1) and 2.35 t.ha^−1^ at the second site (EG2). Very high yields were obtained with Q12 (3.87 t.ha^−1^), Q18 (3.17 t.ha^−1^), and Q29 (3.41 t.ha^−1^).

#### Iran

Only one site has been analyzed considering the lack of observations in the other sites and 11 genotypes were tested (Table 3). They produced an average yield of 0.85 t.ha^−1^ for this site (IN3). Titicaca achieved the highest yield (4.48 t.ha^−1^) in Iran. Note however that some landraces like Q12 (1.03 t.ha^−1^) and Q21 (1.56 t.ha^−1^) have significantly higher values than the arithmetic average of the country.

#### Iraq

Two sites were analyzed for this study and nine genotypes were tested (Table 3) for an average yield of 0.10 t.ha^−1^ at the first site (IQ1) and 0.65 t.ha^−1^ at the second site (IQ2). A value significantly lower than the weighted average yield for all countries was obtained at the two sites and an arithmetic mean for the country also much lower than all countries with only 0.36 t.ha^−1^.

#### Kyrgyzstan

Two sites were analyzed for the study and 3 genotypes were tested (Table 3) with an average yield of 0.90 t.ha^−1^ for the first site (KY1) and 1.03 t.ha^−1^ for the second site (KY2) confirming a value significantly lower than the weighted average yields for all countries at the two sites. Any improved varieties achieved yields higher than 1.24 t.ha^−1^.

#### Lebanon

Three sites were analyzed and 11 genotypes were tested (Table 3). An average yield of 2.7 t.ha^−1^ was obtained at the first site (LB1), 1.15 t.ha^−1^ at the second site (LB2) and 1.82 t.ha^−1^ at the third site (LB3). A value higher than the weighted average yield for all countries was obtained at the three sites. In the first site, landraces Q12 (4.0 t.ha^−1^) and Q29 (4.5 t.ha^−1^) had very high yields which gave a high amplitude to this dataset. This is also the case at the second site where Q18 (2.8 t.ha^−1^) and GIZA1 (2.8 t.ha^−1^) had a high value. This phenomenon is also present at the third site where the two cultivars (GIZA1 and GIZA2) have zero yields as did the landrace Q31. But on the other hand, Q21 (4.3 t.ha^−1^) and Q27 (3.6 t.ha^−1^) achieved very high yields. These important differences between accessions explain a significant dispersion of yields in the three sites within the dataset.

#### Mauritania

Three sites were analyzed for this study and 9 genotypes were tested (Table 3) with an average yield of 0.08 t.ha^−1^ at the first site (MA1), 0.08 t.ha^−1^ at the second site (MA2) and 0.05 t.ha^−1^ at the third site (MA3). The bad arithmetic average with 0.07 t.ha^−1^ was explained by low yields everywhere.

#### Tajikistan

One site was analyzed and only the three improved varieties were tested (Table 3). The study site (TA1) achieved an average yield of 2.13 t.ha^−1^. PUNO and TITICACA achieved good performance with 2.2 t.ha^−1^.

#### Yemen

Three sites were analyzed and 12 genotypes were tested (Table 3). The yield averages obtained were 1.21 t.ha^−1^ at the first site (YE1), 1.14 t.ha^−1^ at the second site (YE2), and 0.72 t.ha^−1^ at the third site (YE3). Thus, one can observe a value higher than the weighted average yields for all countries at the first two sites and an arithmetic average lower than that of all countries at the third site. Q27 achieved the higher yield with 1.9 t.ha^−1^ at YE2, following by Q26 with 1.8 t.ha^−1^ at YE1 and Q1 with 1.7 t.ha^−1^ at YE3.

### Varieties performances across study sites

Figure 3 shows the performance for each genotype across the locations. Most of the genotypes presented higher yields than under Andean conditions in the farming systems examined. Most part of the accessions exhibited huge variations in seed yield data. Considering the large standard deviations observed for each genotype, it cannot be concluded that these accessions have a similar performance across the locations, to define the best one that can be sown with limited risks. The Tuckey test has generated two groups where Q12, Q18, Q21, and Q26 achieved the higher yields (Figure 3, group b) and where Q31 is represented as the lower one (Figure 3, group a); all the other genotypes are intermediaries.

#### Analysis by varietal types

##### Landraces

**Figure 4:**
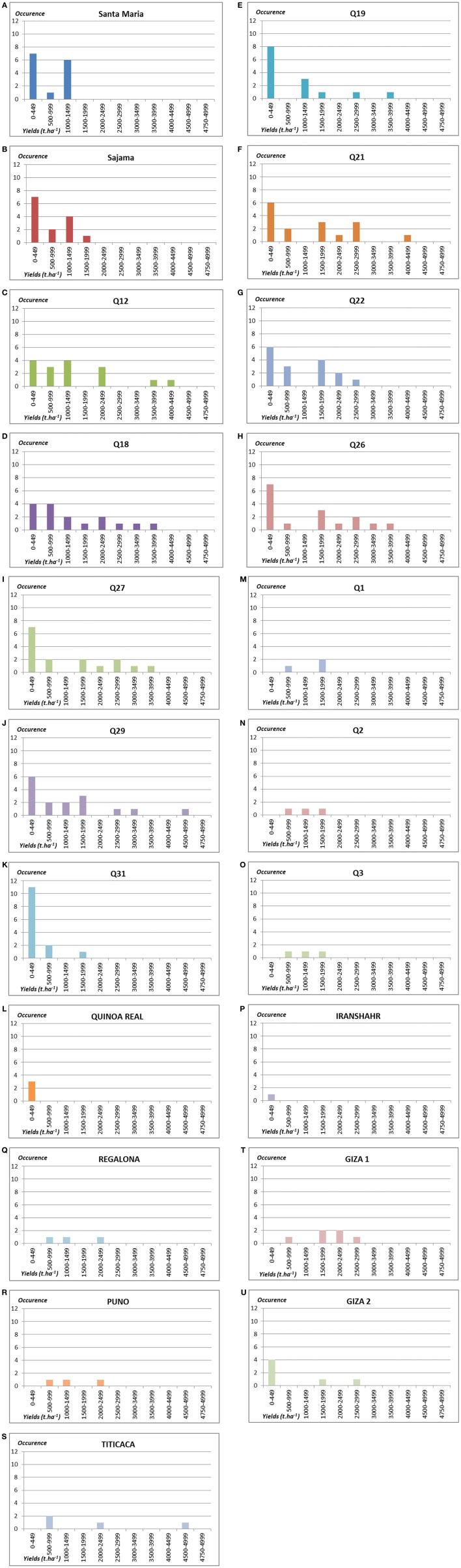
**Number of occurrences of plots by yields' classes in t.ha^−1^ for the 21 genotypes tested: (A) Santa Maria; (B) Sajama; (C) Q12; (D) Q18; (E) Q19; (F) Q21; (G) Q22; (H) Q26; (I) Q27; (J) Q29; (K) Q31; (L) Quinoa real; (M) Q1; (N) Q2; (O) Q3; (P) Iranshahr; (Q) Regalona; (R) Puno; (S) Titicaca; (T) Giza 1; (U) Giza 2**.

In this research project, nine landraces mainly from Chile were tested. When observing the results for each of the study sites (Figures 3, 4), a first dichotomy can be made within landraces. Indeed, we find that three landraces have yields averaging lower than the overall average of 1.07 t.ha^−1^: Q19 (0.89 t.ha^−1^), Q22 (1.05 t.ha^−1^), and Q31 (0.24 t.ha^−1^). The other six landraces present above-average yields: Q12 (1.40 t.ha^−1^), Q18 (1.40 t.ha^−1^), Q21 (1.38 t.ha^−1^), Q26 (1.34 t.ha^−1^), Q27 (1.24 t.ha^−1^), and Q29 (1.20 t.ha^−1^). Nevertheless, the dispersion of data of such landraces is very high and standard deviations are ranging from 1.19 to 1.35.

##### Varieties under development

As part of this study, eight varieties under development or without PVP (Table 2) were tested from different parts of the world (Bolivia, Egypt, Iran, and UAE). After reviewing the results of each of the study sites, they can be separated into two groups (Figures 3, 4). Four of these varieties have average yields below the global average of 1.07 t.ha^−1^: GIZA2 (0.72 t.ha^−1^), SANTAMARIA (0.59 t.ha^−1^), SAJAMA (0.58 t.ha^−1^), and SAJAMA IRANSHAR (0.20 t.ha^−1^). The other four perform better: Q1 (1.30 t.ha^−1^), Q2 (1.13 t.ha^−1^), Q3 (1.13 t.ha^−1^), and GIZA1 (1.88 t.ha^−1^). Moreover, only GIZA1 achieved very promising performance for varieties under development.

##### Improved varieties

For this project, three improved varieties with PVP were also tested. These three varieties have very satisfactory performance levels (see Figure 4) in the few sites where there were tested. Indeed, it may be noted that these three varieties have yield averages much higher than the overall average from 1.37 t.ha^−1^ for REGALONA, to 1.45 t.ha^−1^ for PUNO and to 2.05 t.ha^−1^ for TITICACA, which was the best variety among all the genotypes of panel. PUNO and REGALONA varieties have high uniformity. It is not the case with the variety TITICACA, which presents significant sources of heterogeneity.

## Discussion

### Germination rates and agronomic practices

A very low germination rate was noted for some genotypes tested. This might be due to stocking conditions where high humidity lowers germination quality between seasons (Coulibaly et al., 2015). Large differences in yields were observed among the tested genotypes in the different countries. If global results show that quinoa could be adapted to many environments with higher yields than in traditional cropping systems of Peru or Bolivia, we can state that it is not the case everywhere.

Different reasons could explain the differences, in particular when the seeds of some accessions showed a low degree of germination. Seed quality is the first bottleneck for experimentation because many genotypes were conserved in genebanks for a long time before being sent by plane (submitted to high pressure). Soil preparation generally constitutes the second bottleneck. Before sowing, the soil should be properly prepared to achieve a good *seed-to-soil* contact in the seedbed. The sowing date is another element of this second bottleneck. Preparing the soil under good weather conditions is crucial to obtain this ideal seedbed. All the conditions, including availability of seed accessions, are part of a good installation of the crop in the field avoiding too late sowing. Hot temperature is the third bottleneck and it is very important to plan the sowing date to avoid air temperatures higher than 32°C during the flowering stage, and taking into account that harvest must be done during the dry period. Therefore, the harvested seeds of quinoa need to be well stored at a temperature of less than 15°C under dry conditions to preserve their potential of germination.

### Access to genetic resources

Genebanks are mainly at the origin of development and distribution of our genotypes. However, genebanks should be distinguished as two different entities. We are interested first in genebanks from the Andean region, which can directly provide landraces from their *ex situ* conservation of national germplasm stocks, and secondly in genebanks outside the Andean region such as the USDA which has received seeds of that Andean area. It could also have received seeds from farmers' fields or through collaborations between genebanks. USDA collected landraces on its own account for plant breeding. In addition, USDA provides landraces from its collection to other breeders worldwide who request them in very small quantities (200 seeds) and for a limited number of accessions. While it seems easy to access this genetic material, it can be observed that the distribution is limited due to the limited access to quinoa germplasm and to the limited trade with the Andean countries because of the international regulatory context on seeds: farmers' rights, breeders' rights, phytosanitary rules, and protection of biodiversity against biopiracy. Farmers' varieties (landraces) should be recognized and protected by special agreements between research partner institutions for a particular exchange type, for example through a Standard Material Transfer Agreement (SMTA). The SMTA is a mandatory model for parties wishing to provide and receive material under the Multilateral System of the International Treaty on Plant Genetic Resources for Food and Agriculture but they are often difficult to implement. The Treaty recognizes the contribution that the local farmers of Andean regions, particularly in this case where they are located in centers of origin of crop diversity, have made for the conservation and development of plant genetic resources. Considering and using the SMTA, it gives governments the responsibility for implementing Farmers' Rights, clearly mentioned in its article 9.

Quinoa breeders can be two types: private institutes (Semillas Von Baer, Chile), public institutions (the University of Copenhagen or the CRA-CER Italy) or Non Profit International Organization such as the ICBA. If they have not developed their own collections, they could request access through genebanks, such as the USDA, in limited quantities. They multiply seeds before starting their breeding programs. After that they can benefited from results of this multiplication through the selection of landraces and assign new names. These cultivars or varieties are sold to other organizations, such as FAO that requests quinoa seeds to conduct international tests.

Following this process, there are different possible varietal types that were used in our tests: landraces, improved varieties and varieties under development. However, this genetic material could greatly change over the years and undergo several stages of selection and multiplication in different environmental conditions to those of its original environment. The characteristics of these accessions considered for our trials have probably changed significantly from their Andean nature of origin throughout all these steps.

It is important to note that this process has different pathways for seed diffusion but one can now see that they are related to each other. Being able to see them at separate stages in a continuum, we would see it more as a unique process of distribution and dissemination of genetic diversity of quinoa among different partners and institutions. The main consequence of these constraints on the access to genetic resources of quinoa is that a very low diversity is generally used to conduct trials (Bazile et al., 2016). This happens both, regarding the number of genotypes to be tested, and within the intrinsic genetic diversity of the genotypes for varieties under development and improved varieties that are developed for different environmental conditions (Murphy et al., 2016). These varieties have been selected so that they reduce their genetic heterogeneity which is synonymous to a lower adaptive capacity and contrary to high genetic diversity.

### Relationships with original ecotypes

In order to determine if the characteristics of the sites could be compared to those of the original ecotypes of quinoa, a comparison was made based on three criteria: altitude, latitude and annual rainfall. We noticed that in crossing the variables two by two, we come to systematically identify a group of sites that comes close to the characteristics associated with an original ecotype. In all three cases, the data is always close to the coastal quinoa ecotype that grows at sea level altitude, and latitudes corresponding to more Mediterranean climates. Nevertheless, one cannot completely involve our study sites in this ecotype because their precipitation levels are too far apart. Most study sites have annual rainfall lower than 500 mm, while in the region of origin for this coastal ecotype, from the *Secano Costero* in Chile's central region to the area of Araucanía in the southern Chile, rainfall ranges from 600 to 2000 mm. Landraces from this ecotype, adapted to a level of such precipitation, will not grow easily in the more arid conditions of our study cases, where rainfall rarely exceeds 500 mm, and where additionally 75% of our test sites receive less than 300 mm annually. If access to landraces was improved from the south-central area of Chile, it might be possible to make a screening of accessions to try to find this kind of highly drought resistant genotype. But as access conditions are limited, it is necessary to consider other solutions for quinoa adaptation in new environments.

No experimental site had the same ecological conditions that were observed where the original quinoa ecotypes grew. It is impossible to simultaneously align our three main experimental variables: latitude, altitude and rainfall. However, another solution is available: it is quite feasible to cross two genotypes from two ecotypes with different attributes. For example, using a variety from the ecotype of “Salares” capable of supporting arid conditions (<200 mm rainfall per year) and another variety from the “coastal” ecotype that grows in areas with low altitudes (0–1500 m). A variety capable of growing at low altitude and resistant to low levels of rainfall would be interesting for our study since we find these conditions in most of our test sites. An application of this crossing method for quinoa plant breeding was already done in the past for other attributes, to obtain the variety Regalona, a hybrid between two sources, one close to the equator and the other at southern Chilean regions (Von Baer et al., 2009).

### Discussion of the two strategic objectives for quinoa research related to our initial hypothesis

Plant adaptation in agriculture strategies is essential for building robust production systems that are resilient to the effects of climatic change while strengthening food production for the future (Araus et al., 2008). Choosing new sowing dates or switching the cultivation to a neglected or underutilized existing crop species or variety, may prove to be a very successful move to buffer the cultivation against the negative impact of climatic change. Leveraging the potential of genetic diversity along with the deployment of improved best practices, are relatively inexpensive measures affordable by farmers, especially when accompanied by adequate extension work. On the other end, conventional plant breeding and crop's improvements are more expensive and require more technology investment for pursuing the goal of greater productivity. For example, the increase in food production in recent years has been mostly achieved considering the expansion of irrigated lands. Water use efficiency appears now as a key factor to consider from another option of view the effects of climatic change (Seckler et al., 1999). In fact, assessment of new crops and varieties diffusion of traditional and improved crop varieties needs to take more into account agronomic practices suitable and sustainable for specific ecosystems.

In addition, during the twentieth century, plant breeding for major crops has contributed to the higher increase of crop productivity for the mentioned species. But at the same time, this successful story in crop yields was limited in more recent years with a stabilization of yields for wheat and rice (Conway and Toenniessen, 1999). The situation regarding crop yields nowadays is still more complex and local context of cultivation have to be more considered for agronomic practices but also for their social and cultural dimensions (Bazile, 2015).

#### Maximizing yields

National breeding programs, which are primarily concerned on how to moderate food insecurity, may modify some aspects of the agricultural intensification derived from the Green Revolution strategy (Annicchiarico, 2002) in order to select and to produce improved germplasm, socio-economically convenient, capable of maximizing the agricultural potential of specific areas and farming systems in marginal areas, and of minimizing the occurrence of crop failures or very low yields in unfavorable years. The ultimate goal of this quinoa program is to achieve adaptation and yield stability targets, especially by using variety material with increased tolerance to prevailing biotic and abiotic stresses.

As part of our study, yield maximization strategies echoes our first hypothesis and it is important to remember: “*Within the high genetic diversity of the species, it is possible to identify the most suitable variety for each study site*.” Based on the outcome of the studies, this first hypothesis was confirmed since there are always one or more genotypes of superior performance in one site when compared to other sites, reflecting their excellent adaptability for higher production under these particular ecological conditions. However, we should ask whether this strategy to maximize productivity, considering the performance as yield per hectare, would not be somewhat inappropriate to the context of the study in terms of food security. In addition, the tested environments are all of them located in marginal areas under extremely dry conditions and sometime saline soils that cannot permit many choices of crops that support those. This intensive agricultural model would be relevant if we sought to obtain or to increase profits in the short term, for a major sale of production surplus, for example. In our context, we are not really focusing on production intensification but rather to contribute to the food security of local populations, mostly in a subsistence farming context, that is achieving sustainable productions also in difficult areas and climate conditions. Moreover, it is important to focus on the environmental risks linked to this agricultural conventional model more based on technology intensification than on ecological intensification. As part of a high production target, we often use a significant amount of inputs, such as pesticides or chemical fertilizers, which increase the level of pollution of the local environment and present health risks to populations (Caron et al., 2009). Given the limited investment capacity of small-scale farmers in these countries, the potential yield of these varieties of quinoa will never be achieved without an initial capital contribution, in terms of expenses related to inputs, to be deducted from the final performance available for sale or supply.

#### Optimization for stability of the food production

From an ecological point of view, adaptation is considered as a process where the *adaptedness* is the level of adaptation of plant material to a given environment, and *adaptability* is the ability to present good adaptedness in a wide range of environments (Tigerstedt, 1994). For plant breeders, the two terms refer to a condition rather than a process. It shows the ability of plant material to be high-yielding with respect to a given environment or given conditions (Gallais, 1992). For agronomist, the accepted definitions need to consider more changes across time and space. Efforts may be developed for investigating crop genetic diversity in space and time under plant breeding programs, in relation with farmers and agronomists, for more sustainable crop production (Fu, 2015).

After demonstrating that a maximization strategy is not necessarily the most appropriate to our project, it is necessary to take a different look to find an alternative pathway for quinoa testing and introduction in new environments. We had already mentioned another strategy, raising the importance of a lasting and stable food system for the populations of the countries studied. This vision of stability, as an important dimension of food security, is embodied by our second hypothesis of work and concern directly the adaptability in a wide range of environments: “*To meet the various uncertainties related to global change in each region, yield stability of one variety across all study sites represents an important indication of genotype suitability and decreased risks when cultivating it any other given area*.” We wanted to demonstrate, whether one or more varieties have a level of stable and efficient performance across all or most of our study sites. An average grain yield of all varieties in each location has been used to compare the performance of each variety. All varieties were listed with a higher yield than the average yield of the site. Finally, the varieties were classified based on the number of times each variety appeared higher than the average of the different study sites. Six genotypes (Q12, Q18, Q21, Q22, Q26, and Q27) have an occurrence of up to five and present good yield potentials with great stability across sites. This result was confirmed with a good ponderation by the number of sowing sites. This gives us another perspective for less considered varieties in the trials (Q22, Q27) considering the Tuckey's method used for comparisons. The improved varieties, Puno, Titicaca (and also Regalona) performed with more than 75% but they need to be tested under more various conditions considering their good yield production, with the first and third average yield among all the genotypes.

#### Strategic choices and their consequences

As previously mentioned, the main objective of the FAO programme was primarily to explore ways to strengthen with quinoa cultivation the food and nutritional security of the countries involved. Our goal in fact was to achieve food security at different scales (local, national, and global). In addition, we have seen that the nutritional properties of quinoa are exceptional, especially due to its high protein levels correlated with a good balance of essential amino acids for humans. A slight amount of these grains in the daily diet would provide an interesting contribution in vegetable protein for local people which have no access to other sources of proteins.

Therefore, these two agricultural models, maximizing vs. optimization, do not tend toward the same goals and do not have the same impact on food security. They are incompatible, although they may coexist within a country in different geographical areas or for differentiated groups of farmers. As explained before, we would privilege more the food security of the countries through a yield optimization strategy that corresponds to the overall objective of the project supported by FAO for achieving adaptability and stability as requested by governments of these countries (Power, 1999). Therefore, the choice of a model that implies a specific kind of variety corresponds to the main objective. In our case, it should be preferable to choose genotypes that do not necessarily have higher yield levels. These genotypes must produce a higher average yield measured with a constant, regardless of its stability production and adaptability in marginal environments (Bazile and Weltzien, 2008; Choukr-Allah et al., 2016).

However, one should not completely ignore the model of maximizing returns (Fasoula, 2012). In fact, this strategy could be considered in those countries assuming a long term insertion of quinoa in the local agricultural landscapes and cropping systems. For example, some farmers in Morocco with larger areas are already able to take a greater risk with capital investment (equipment and inputs) to target the maximum potential of improved varieties of quinoa (Benlhabib et al., 2015).

### Conclusion: looking for an insertion in local cropping systems

In this research, the study tested two hypotheses in order to demonstrate the adaptability of quinoa outside the Andean area. One point of discussion through different perspectives must the sustainable establishment of an alien species in a new agricultural environment. The crop introduction has to move from the stage of agronomic testing to that of assessing in practice whether quinoa can take place easily and sustainably in local farming systems around the world. Focusing on major crops cultivated in project partner countries to better know their crop cycles, we analyzed the potential inclusion of quinoa in agricultural calendars. Three groups of countries differ significantly: first when quinoa is considered a winter crop (Lebanon, Egypt, and Yemen), second when it operates as a spring crop (Kyrgyzstan and Tajikistan) and finally where it is planted as a fall crop (Algeria, Iraq, Iran, and Mauritania). Such disparities are interesting to study more in depth, considering quinoa crop introduction in a geographical area in appearance with somewhat similar climatic conditions. This consideration underlines that climate data is the main lever of action to decide the sowing date and to understand the best position in the crop calendar for water use efficiency and to avoid high temperatures at the flowering period.

The main conclusion of this study is that we were able to measure and confirm the great adaptability of quinoa in new areas with very different climatic conditions than those of the area of origin of this crop. In each of the sites studied, we were able to identify one or more genotypes with a high performance level validating the hypothesis 1 of yield quality performance. High yield potentials in single locations were shown by some genotypes like Q29 (LB1), Titicaca (IN3), Q21 (LB3), Q12 (LB1), Q29 (EG2), Q27 (LB3), Puno (TA1), Q1 (YE3). Considering yield optimization within a long term vision, some genotypes maintain a good level of stable performance regardless of the specific site conditions in which they were tested. This result allows us to also validate our second hypothesis of yield stability across sowing sites. The genotypes Q12, Q18, Q21, and Q26, averaged the best yield across 16 sites. In an agroecological perspective, quinoa biodiversity could be interesting to diversify cropping systems in order to stabilize food production.

Quinoa should be considered for diversifying cropping systems for food security and it should not be incorporated as part of an already busy period of the crop calendar cycle, and then competing with other cultures that have already demonstrated their good performances in these areas. In addition, one must not focus solely on the agricultural side of the introduction of quinoa in a local agro-ecosystem. The social component remains an important part of the adaptation of exotic species and its appropriation by the new populations. The food system of the study area may be upset with the introduction of a new food. Local populations will have to integrate quinoa in their diets and they will need to be prepared and technically and culturally accompanied to handle the new grains. There is still doubt on this point because, although quinoa has exceptional nutritional properties, development of attractive and culturally acceptable food recipes will need to be developed to reach out to both rural and urban consumers. This work will be an important endeavor to take into account when considering the introduction of quinoa cultivations outside the Andean region.

## Author contributions

MA, DB, JB, LH, IM, OM, MO, NS, AS, DS, and KM have established the multi-local trials across the different countries and have collected the data from the field. DB and CP have given a permanent technical assistance and some specific orientations for the experimental plan during the implementation of the trials and their monitoring. DB conducted the analysis on results from the multi-local trials and AV contributed to the standardization of the dataset and to the first step of statistical analysis. SP collaborated with AV and had a global overview of the writing process.

### Conflict of interest statement

The authors declare that the research was conducted in the absence of any commercial or financial relationships that could be construed as a potential conflict of interest. The views expressed in this paper are those of the authors and do not necessarily reflect the views or policies of FAO.
