# Supplementary figures and images for: Worldwide Evaluations of Quinoa: Preliminary Results from Post International Year of Quinoa FAO Projects in Nine Countries
Source: Front Plant Sci. 2016 Jun 21;7:850. doi: 10.3389/fpls.2016.00850 (PMC4914551; doi:10.3389/fpls.2016.00850)

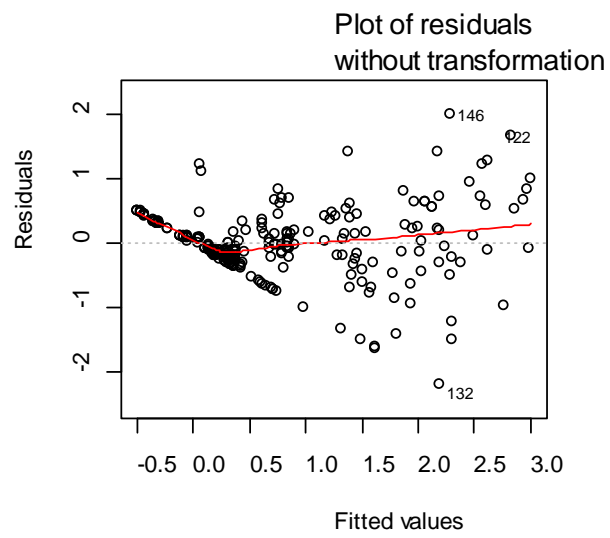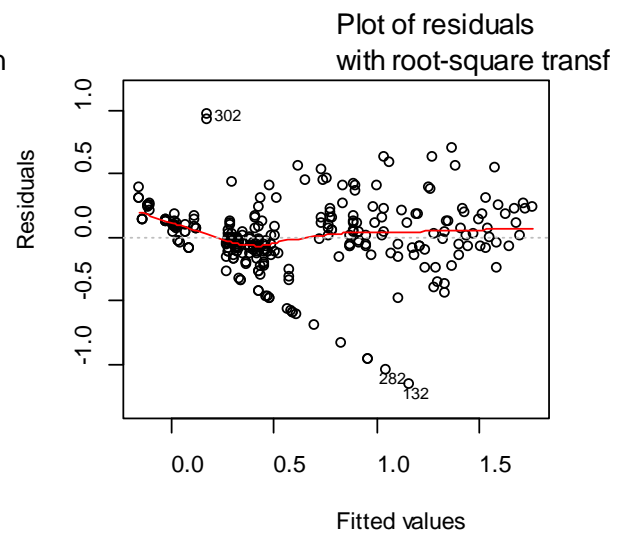

Supplement: Supplementary file 1 [file DataSheet1.PDF]
